# Senescence: No country for old cells

**DOI:** 10.1016/j.bj.2023.100697

**Published:** 2023-12-29

**Authors:** Jan Martel, David M. Ojcius, John D. Young

**Affiliations:** Center for Molecular and Clinical Immunology, Chang Gung University, Taoyuan, 33302, Taiwan; Center for Molecular and Clinical Immunology, Chang Gung University, Taoyuan, 33302, Taiwan; Department of Biomedical Sciences, Arthur A. Dugoni School of Dentistry, University of the Pacific, San Francisco, CA, 94103, USA; Immunology Consortium, Chang Gung Memorial Hospital at Linkou, Taoyuan, 333, Taiwan; Chang Gung Biotechnology Corporation, Taipei, 105406, Taiwan

## 1 Introduction

**Fig. 1:**
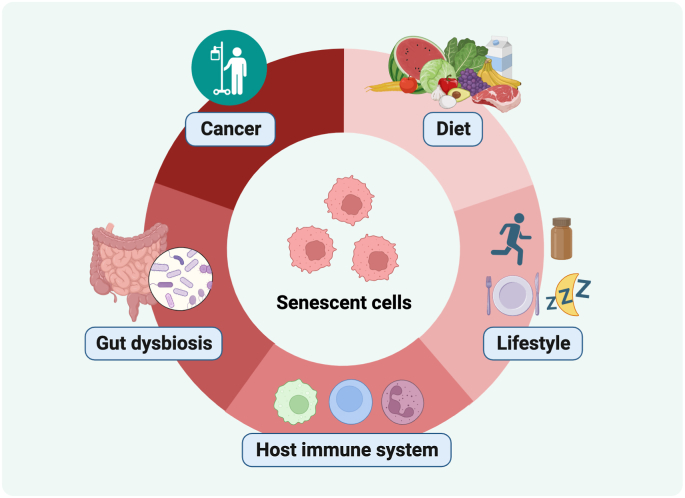
Factors affecting senescence, aging and longevity that are covered in this special issue.

Human health requires homeostasis and coherence to respond to stress and synchronize physiological functions. This state of balance is gradually lost during aging when senescent cells accumulate and persistently secrete cytokines that promote systemic inflammation and affect various organs. The immune system is well trained to track and kill senescent cells, but immune cells become old and eventually fail to keep up. In this special issue on senescence, seven original contributions present recent advances related to the roles and characteristics of senescent cells and how senescence, aging and longevity are influenced by cancer, gut dysbiosis, immune cells, lifestyle and diet [Fig. 1].

## 2 Body size, longevity and cancer

Studies suggest that tall humans have higher all-cause and cancer mortality and a shortened lifespan than people of shorter stature [1]. This observation may be due at least in part to increased growth hormone signaling which promotes growth mechanisms at the expense of repair [2]. However, when various animal species are compared, there is no apparent increased incidence of cancer in species showing larger body size and longer lifespan, an observation known as “Peto's paradox” [3]. Perillo and colleagues studied the immortalization and replicative capacity of fibroblast cell strains from 17 mammalian species and observed a negative correlation between immortalization and species body mass [4]. The authors conclude that evolution has led to the development of stringent mechanisms to preserve genetic stability and prevent cancer in species that developed a large body weight. In a companion review article [5], the authors summarize the mechanisms involved in preventing cancer and increasing cellular longevity in species with large body mass.

Mirisola and Longo studied the cellular pathways involved in stress resistance and longevity in yeast [6]. They identified mutations in several phosphatases that reverse the effects of constitutively activated Ras on stress sensitization and longevity [6]. Given that mutations and alleles of Ras are associated with human tumors and pro-aging pathways, the identified proteins represent potential targets to treat cancer and extend longevity.

## 3 Aging and the immune system

Giannoula et al. describe the complex interactions between senescent and immune cells during the aging process [7]. It becomes apparent that excessive production of senescent cells and insufficient elimination of such cells by the immune system contribute to the disruption of physiological functions observed in aging. The authors describe how damaged or senescent cells normally excrete harmful cytosolic DNA encapsulated by exosomes [8], but these can activate surrounding cells and induce inflammation by serving as damage-associated molecular patterns (DAMPs). Natural killer cells have been shown to display senolytic properties and eliminate senescent cells, reducing organ damage and liver fibrosis [9]. The authors propose that various new treatments, from engineered chimeric antigen receptor (CAR)-T cells to rejuvenation of the immune system with bone marrow transplantation, are promising strategies to reduce the accumulation of senescent cells in the body [7].

## 4 It's all about (life)style!

Martel and colleagues argue that many lifestyle interventions that are safe, cheap and effective are already available to reduce the burden of senescent cells [10]. Physical exercise can reduce the accumulation of senescent cells in various organs [11,12], but it is important not to overdo it since strenuous exercise can induce detrimental effects via hormesis [10,13]. Drastic nutritional recommendations can be hard to follow, but a fasting-mimicking diet followed for four days and carried out bi-monthly can have a rejuvenating effect on the immune system [14], which may help reduce the number of senescent cells that persist in the aging body. Phytochemicals from citrus fruits (quercetin), green tea (polyphenols), and strawberries (fisetin) also produce anti-senescence effects [15]. Quercetin (in combination with dasatinib) and fisetin are currently being tested in clinical trials to determine if they can reduce the detrimental effects of senescent cells in humans. Many effects of phytochemicals are mediated by the gut microbiota [16] and additional studies are needed to examine the effects of dietary fiber, mushroom polysaccharides, and probiotics on the burden of senescent cells in the body. But we should not lose sleep over senescence since sleep deprivation increases markers of DNA damage, senescence and inflammation [17], thereby contributing to the development of chronic diseases and promoting aging.

## 5 Macronutrient metabolism and aging

Nehme and colleagues explain how macronutrients from the diet may influence the aging process and longevity [18]. Low-glycemic diets can extend the lifespan of mice [19], possibly by reducing reactive oxygen species (ROS) and inflammation, while preventing the reduction in nitric oxide (NO) and sirtuin activity observed in aging [18]. For proteins, the amount is important, with low-protein diets (less than 10 % of calories from proteins) being associated with a reduction in all-cause and cancer mortality, whereas the situation is reversed for people over 65 who should increase their protein intake [20]. The source of proteins is also important as animal proteins are associated with increased mortality from cardiovascular disease compared to plant proteins [21]. While excessive fat intake is widely recognized to be detrimental to health and aging, Nehme et al. note that the quality of dietary fats and the balance with other nutrients may play a more critical role on health outcomes than simply the amount of fats consumed. The authors conclude that calorie restriction, ketogenic diets, and intermittent fasting also represent ways to delay aging and reduce the senescence burden [18].

## 6 Link between immunosenescence, the gut and kidneys

Lee et al. review the link between disruption of immune functions during aging and possible effects on the gut and kidneys [22]. Patients with chronic kidney disease (CKD) show impaired innate and adaptive immune functions. For instance, subjects with end-stage CKD show increased levels of CD14^+^CD16^+^ circulating monocytes and higher expression of Toll-like receptor-4 (TLR4) in neutrophils and monocytes [23]. CKD patients also show signs of thymic function decline and a shift of B and T lymphocytes towards a senescent and exhausted phenotype [22]. Gut dysbiosis is linked with increased gut permeability and the production of bacterial metabolites such as p-cresol, indole, and trimethylamine (TMA) that can lead to kidney toxicity [22]. While it is still not clear which factor comes first—aging, impaired kidney function, immunosenescence, gut dysbiosis, or chronic psychological stress—many potential suspects, locations and phenomena have been identified … Who's up for a game of Clue?

Some of the studies and reviews in this special issue suggest that old senescent cells will have no clean getaways [24] and may soon have to face a new arsenal of anti-senescence weapons …

## Disclaimer statement

J.M., D.M.O., and J.D.Y. are named on patents held jointly by Chang Gung University and Chang Gung Biotechnology related to the preparation and use of dietary supplements. J.D.Y. is Chairman of the Board of Chang Gung Biotechnology.
